# Enterovirus-Human Rhinovirus as a Leading Cause of ARDS in a Liver Transplant Recipient

**Published:** 2019-02

**Authors:** Meysam Yousefi, Seyed Alireza Nadji, Behrooz Farzanegan, Seyed Mohammad Reza Hashemian, Majid Marjani, Afshin Moniri, Pedram Javanmard, Payam Tabarsi

**Affiliations:** 1 Research Center of Tropical and Infectious Diseases, Kerman University of Medical Sciences, Kerman, Iran,; 2 Virology Research Center (VRC), National Research Institute of Tuberculosis and Lung Diseases (NRITLD), Shahid Beheshti University of Medical Sciences, Tehran, Iran,; 3 Tracheal Diseases Research Center, NRITLD, Shahid Beheshti University of Medical Sciences, Tehran, Iran,; 4 Clinical Tuberculosis and Epidemiology Research Center, NRITLD, Shahid Beheshti University of Medical Sciences, Tehran, Iran

**Keywords:** Acute respiratory distress syndrome (ARDS), Enterovirus-Human Rhinovirus, Immunosuppression, Transplantation

## Abstract

A 35- year- old man with a prior history of liver transplantation 18 months ago was admitted to our Intensive Care Unit (ICU) with fever and worsening dyspnea and was diagnosed with severe pneumonia leading to Acute Respiratory Distress Syndrome (ARDS). He had a prolonged hospitalization and was treated with empiric broad spectrum intravenous antibiotics, oseltamivir, trimethoprim/sulfamethoxazole, and subsequently caspofungin and ganciclovir.

Blood, nasopharyngeal, as well as Bronchoalveolar Lavage (BAL) culture and Polymerase Chain Reaction (PCR) were negative for all viral, bacterial, and fungal causes of pulmonary infection except Enterovirus-Human Rhinovirus (EV-HRV) that was positive with high titers on BAL and swab specimens. Consequently, the diagnosis of EV-HRV pneumonia complicated by ARDS was established. The patient gradually improved and was discharged from the hospital after 3 weeks. This report highlights EV-HRV as a cause of ARDS in immunocompromised adults.

## INTRODUCTION

Enterovirus-Human Rhinovirus (EV-HRV) are single stranded RNA viruses of the picornavirus family and are the most common causes of colds in children and adults (1). EV-HRV commonly causes mild upper respiratory tract illnesses; however, in rare instances they can cause severe and potentially fatal conditions such as Acute Respiratory Distress Syndrome (ARDS).

In the past, these viruses were under-recognized as a significant pathogen in immunocompromised patients, but several recent prospective studies using nucleic acid testing have clearly demonstrated that Rhinoviruses are probably the most common respiratory viral pathogen in transplant recipients (2–7).

In this article, we present a young man who developed ARDS secondary to EV-HRV infection.

## CASE SUMMARIES

A 35–year-old man with a prior history of liver transplantation 18 months ago was admitted to the Intensive Care Unit (ICU) of our hospital with respiratory failure.

He presented with persistent fever, cough, and shortness of breath which had first began two months ago after returning from a trip to Karbala, Iraq. Despite prolonged outpatient treatment including antibiotic therapy during this period, there was progression of symptoms which required hospital admission and subsequent transfer to our center.

His past medical history was significant for ulcerative colitis and Primary Sclerosing Cholangitis (PSC) diagnosed 16 years ago leading to liver transplantation 18 months prior to admission. After transplantation, there were no major complications to this point and his medications consisted of prednisone (5 mg), mycophenolate (6 tablets) and tacrolimus (1 mg) per day.

At the time of admission, he was alert but febrile and tachypneic. His blood pressure was 100/60 mmHg, respiratory rate 35/min, temperature 38.5 degrees celsius, O_2_ sat 85%, PaO_2_ 45 mmHg, and FiO_2_ 0.20 (PaO_2_/FiO_2_ ratio: 225).

Complete blood count revealed white blood cells 14,300/mm^3^ with a left shift, platelets 38,900/mcl, SGPT 26U/L, SGOT 23U/L and procalcitonin of 0.72ng/ml.

**Figure 1.**
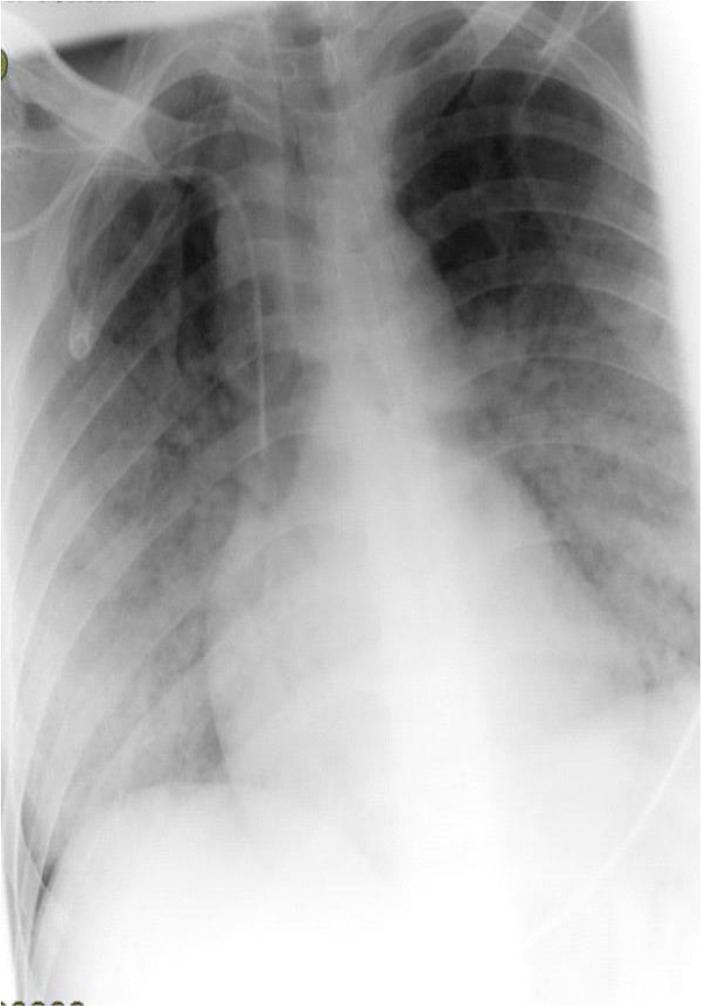
Chest X-Ray showed diffuse opacities

Rales were heard in both lungs. Chest Radiography revealed pulmonary vascular congestion and bilateral alveolar infiltration (Figure 1).

**Figure 2.**
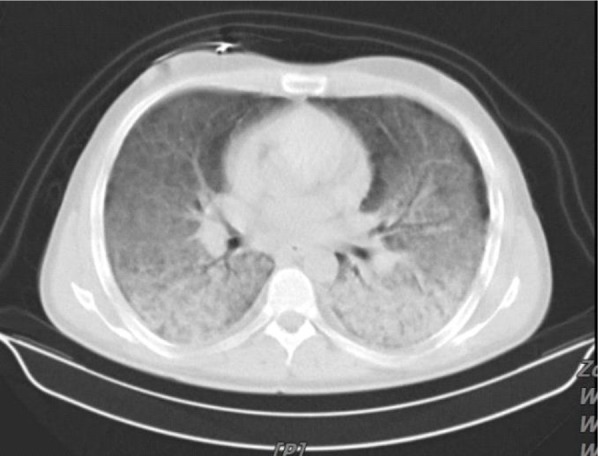
CT- Scan of the chest showed bilateral alveolar infiltration with Air Bronchogram and ground-glass opacification

CT- Scan of the chest showed bilateral alveolar infiltration with Air Bronchogram and ground-glass opacification (Figure 2).

Transthoracic echocardiogram (Echo) was normal, and duplex ultrasound scan of the lower extremities was negative for thrombosis.

Non-invasive ventilation was started with concomitant Meropenem (1g q3h), Vancomycin (15 mg/kg q12h), Levofloxacin (750 mg q24h), Oseltamivir (75 mg q12h) and high dose trimethoprim/sulfamethoxazole. Concurrently, a pharyngeal swab for Influenza and other viruses, blood cultures, CMV PCR, Serum galactomannan, 1,3-BD Glucan, and cryptococcal antigen was sent (Table 1).

**Table 1.** Laboratory data

Swab for influenza	Neg
CMV PCR VIRAL load	2466 copy/ml
Blood culture	Neg
Serum galactomanan	Neg
1,3-BD Glucan	90
Cryptococcal Ag	Neg

After 48 hours of treatment, there was no significant clinical improvement; therefore, Caspofungin and ganciclovir were added to the previous treatment regimen. Furthermore, bronchoscopy was performed with the multiplex amplified nucleic acid testing of the Bronchoalveolar Lavage (BAL) fluid positive only for rhinovirus with high titers, consistent with the pharyngeal swab. All other viral, bacterial, and fungal organisms were negative.

The diagnosis of Rhinovirus pneumonia causing ARDS was established. The patient gradually improved and discharged from hospital after 3 weeks.

## DISCUSSION

Enterovirus-Human Rhinovirus (EV-HRVs) are single stranded RNA viruses of the picornavirus family that commonly cause mild respiratory tract illness. However, in some cases they can cause severe and potentially fatal conditions such as aseptic meningitis, encephalitis, myocarditis, viral pneumonia, and ARDS (8–11).

The severity of respiratory symptoms in EV-HRV infection depends on the production of numerous pro-inflammatory cytokines and chemokines such as (interleukin) IL-1, IL-6 and IL-8 (12). These pro-inflammatory molecules cause airway inflammation.

ARDS is characterized by diffuse inflammation of the lung leading to severe respiratory distress and hypoxemia refractory to oxygen therapy. ARDS is a clinical phenotype that can be triggered by various etiologies including infection, trauma, and sepsis. It is associated with high mortality rates ranging from 25 to 58% (13–15).

Rhinovirus commonly affects the pediatric age group, patients with chronic lung disease, and those who might be immunocompromised including patients with HIV, leukemia, and organ transplants.

Very few reports of EV-HRV causing severe ARDS in adults have been described in the literature. The pathogenesis of the severe respiratory illness caused by Rhinoviruses is not clear (11, 15). Early identification of EV-HRV infection using multiplex PCR techniques can limit the use of antimicrobial agents in such patients. As demonstrated in this case, it is important to consider EVHRV in the differential diagnosis of pneumonia causing severe ARDS in immunosuppressed patients.

## CONCLUSION

It should be kept in mind that human rhinovirus infection may cause severe lower respiratory tract infection and ARDS.
